# Regulation of B Cell Differentiation by Intracellular Membrane-Associated Proteins and microRNAs: Role in the Antibody Response

**DOI:** 10.3389/fimmu.2015.00537

**Published:** 2015-10-26

**Authors:** Zheng Lou, Paolo Casali, Zhenming Xu

**Affiliations:** ^1^Department of Microbiology and Immunology, School of Medicine, The University of Texas Health Science Center, San Antonio, TX, USA

**Keywords:** B cell activation and differentiation, plasma cell, memory B cell, intracellular membrane associated proteins, endosome, lysosome, autophagosome, microRNA

## Abstract

B cells are central to adaptive immunity and their functions in antibody responses are exquisitely regulated. As suggested by recent findings, B cell differentiation is mediated by intracellular membrane structures (including endosomes, lysosomes, and autophagosomes) and protein factors specifically associated with these membranes, including Rab7, Atg5, and Atg7. These factors participate in vesicle formation/trafficking, signal transduction and induction of gene expression to promote antigen presentation, class switch DNA recombination (CSR)/somatic hypermutation (SHM), and generation/maintenance of plasma cells and memory B cells. Their expression is induced in B cells activated to differentiate and further fine-tuned by immune-modulating microRNAs, which coordinates CSR/SHM, plasma cell differentiation, and memory B cell differentiation. These short non-coding RNAs would individually target multiple factors associated with the same intracellular membrane compartments and collaboratively target a single factor in addition to regulating AID and Blimp-1. These, together with regulation of microRNA biogenesis and activities by endosomes and autophagosomes, show that intracellular membranes and microRNAs, two broadly relevant cell constituents, play important roles in balancing gene expression to specify B cell differentiation processes for optimal antibody responses.

## Introduction

B lymphocytes are critical to immunity by mediating production of neutralization antibodies to infectious pathogens and tumor cells (1). They develop through several highly regulated steps, first as pro-B cells and then pre-B cells in the bone marrow, in which immunoglobulin (Ig) V(D)J DNA recombination occurs, and subsequently as immature B cell stages and then mature B cells in the periphery. V(D)J recombination gives rise to a highly diverse repertoire of the B cell receptor (BCR) for antigens. Unlike immature B cells, which express predominantly IgM-containing BCRs, mature naïve B cells express high levels of IgD on their surface. In secondary lymphoid organs (e.g., spleen and lymph nodes), B cells are organized into the follicular or marginal zone areas (2), in part due to their BCR signaling differences.

Upon antigen encounter, B cells are activated in a T cell-independent or T cell-dependent manner. In T-independent antibody responses and the early T-independent phase of T-dependent antibody responses, B cells differentiate upon dual engagement of BCR (e.g., by repetitive antigenic ligands) and an innate immune receptor, such as a toll-like receptor (TLR) (3). In T-dependent antibody responses, specific B cells are induced through cognate interaction, and engagement of CD40, which is constitutively expressed on B cells, by trimeric CD154 expressed by specific T helper (T_H_) cells (4). Activated B cells differentiate in germinal centers, the newly formed specialized microenvironment within secondary lymphoid organs (5). They undergo class switch DNA recombination (CSR) in the Ig heavy chain (IgH) locus to switch their BCR from IgM or IgD to, depending on eliciting stimuli, IgG, IgE, or IgA, which endows an antibody with different biological effector functions without changing antigen specificity (4). B cells also undergo somatic hypermutation (SHM). This inserts point-mutations into Ig V region DNA, thereby providing the substrate for positive selection of antibody mutants with higher affinity to the antigen (6). Finally, B cells differentiate into plasma cells, which secrete large amounts of antibodies, or memory B cells, which can be re-activated for amnestic antibody responses upon second challenge by the same antigen (7–10). Memory B cells and plasma cells are long-lived and play an important role in the protection against re-exposure to microbial and other antigens.

B cell CSR/SHM and differentiation into plasma cells and memory B cells are tightly regulated. Dysregulation of these processes can lead to immune deficiencies, autoimmunity, or B lymphomagenesis. In this review, we will focus on how B cell differentiation is regulated for effective antibody responses by intracellular membranes, particularly the emerging functions of endosomes and autophagosomes. We will also emphasize the induction of proteins associated with these intracellular membranes and the role of these proteins in signaling and induction of gene expression. Furthermore, we will summarize the evidence of the epigenetic modulation of expression of these proteins by microRNAs and the consequence to B cell differentiation – readers are referred to other articles that have extensively reviewed the regulation/dysregulation of immune functions by microRNAs (11–15). Finally, we will discuss the notion that microRNA activities are reciprocally regulated by intracellular membranes, as part of the controlling mechanisms that fine-tune gene expression and buffer molecular aberrancies to ensure the specificity of B cell differentiation and functions.

## B Cell Regulation by Intracellular Membrane-Associated Proteins

### Intracellular Membrane Structures and Associated Proteins

**Figure 1:**
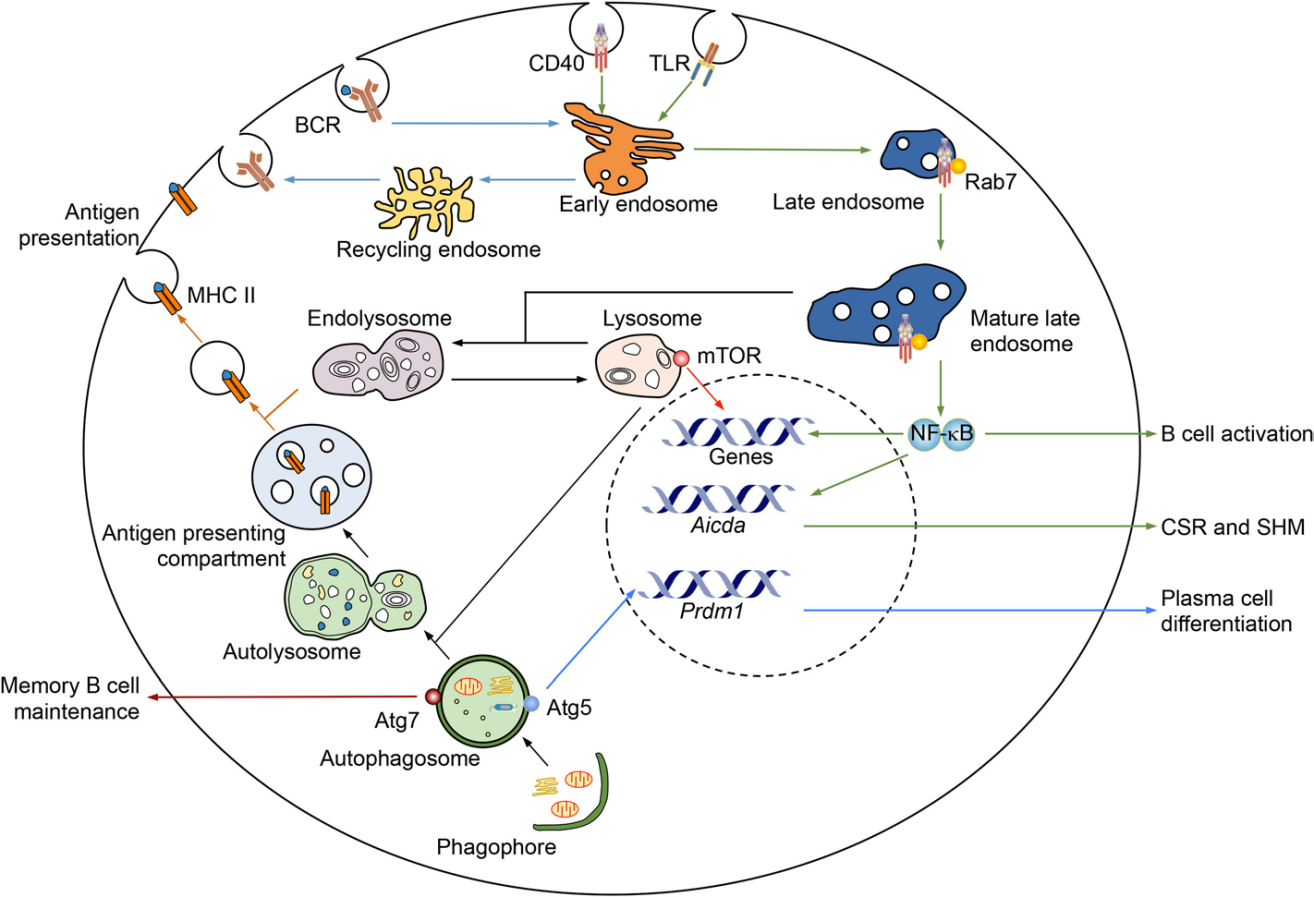
**Regulation of B cell differentiation and functions by intracellular membranes and associated proteins**. Several B cell processes are regulated by endosomes, lysosomes, and autophagosomes. Upon antigen-triggered internalization, BCR is sorted by early endosomes to either recycle back onto the cell surface or go through antigen processing, which are mediated by endolysosomes, for the MHC II-dependent antigen presentation. Signaling receptors, such as CD40 (as depicted) and TLRs (both surface TLRs and intracellular TLRs), can be internalized and/or sorted by early endosomes to localize to mature late endosomes, as marked by Rab7. A Rab7-dependent process would stabilize the interaction of such receptors and their adaptors (e.g., CD40 and TRAF6), thereby promoting sustained signaling, such as NF-κB activation, for induction of genes important for B cell activation and CSR/SHM, e.g., AID. Lysosomes, as transformed from and maintained by mature late endosomes, can recruit mTOR, which plays an important role in B cell activation. Autophagosomes are important for memory B cell maintenance, i.e., through Atg7, and plasma cell differentiation and survival, e.g., through Atg5. They can also fuse with lysosomes to become autolysosomes, which then transform into a specialized compartment for antigen presentation.

Endosomes are internalized lipid vesicles containing extracellular molecules and cell surface components, including fluid, solutes and their carriers, lipids, membrane proteins, and receptor–ligand complexes (16). They regulate various cellular processes by sorting, processing, recycling, storing, and degrading these cargos. Early endosomes, which have tubular and vacuolar domains, are the main sorting station of internalized cargos, most of which are recycled back to the plasma membrane directly or through recycling endosomes (Figure 1). Late endosomes form from the vacuolar domains of early endosomes, with many changes in the lipid and protein contents. They mature upon moving from the peripheral cytoplasm to the perinuclear area, where mature late endosomes fuse with each other and eventually fuse with lysosomes or pre-existing hybrid organelle endolysosomes (Figure 1). Endosome maturation is regulated by a group of Rab (Ras-related in brain) small GTPases (17). Rab5 is a defining component of early endosomes through various stages, and regulates the conversion of early endosomes to late endosomes (17). The GTP-bound form of Rab5 (Rab5-GTP) recruits Rab7 and is then converted to Rab5-GDP, which dissociates from the membrane (18, 19). Rab7, upon recruitment, can auto-activate to bind GTP and suppress Rab5 binding to GTP (20). Rab7-GTP recruits its own effectors, including RILP, which connects late endosomes to dynein motors for trafficking, and components of the HOPS complex, which promotes late endosome fusion with lysosomes (21, 22). Lysosomes are the terminal degradative compartments. These have a luminal pH of 4.6–5.0 and contain hydrolases that digest the cargo (17). They can also fuse with autophagosomes to form autolysosomes, which carries out degradation of extracellular and intracellular components.

Macroautophagy (referred here as autophagy) is a process that degrades cytoplasmic compartments and organelles and is underpinned by the formation of autophagosomes, which are enclosed double-membrane structures (23). Autophagy was first characterized as a cellular process to counter stress, e.g., by mediating degradation of cytoplasmic organelles/proteins in starved cells for the generation of energy and macromolecules to maintain cell viability (24). Autophagosomes, however, can be induced under physiological stress-free conditions, likely through slightly different pathways, and play important roles in immunity (25, 26). Their induction starts from activation of a protein complex containing ULK1 and the autophagy-related gene (Atg) 13, and formation of a phagophore, a double-membrane structure (Figure 1). The nucleation of phagophores requires activation of the class III phosphatidylinositol 3-kinase (PI-3K) complex consisting of the VPS34 (vacuolar protein sorting 34), which is activated by a complex containing Beclin 1, VPS15, and Atg14 (24). Phosphorylation of ULK1 and Atg13 by the mTOR kinase, which regulates B cell differentiation and CSR (27, 28), inhibits autophagy activation. Inhibition of mTOR, e.g., by rapamycin, promotes autophagosome formation (29). Autophagosomes form after the expansion and final closure of phagophores, and then fuse with either mature endosomes to form amphisomes or lysosomes to form autolysosomes, in which luminal cargos and the inner membrane are degraded (Figure 1) – amphisomes can also fuse with lysosomes to form autolysosomes.

### Antigen Presentation

In T-dependent antibody responses, B cells uptake antigens through the BCR, process it, and present it through the major histocompatibility complex II (MHCII) to cognate CD4^+^ T_H_ cell TCR to prime T_H_ cells. These, in turn, will prime B cells for full activation, proliferation, and differentiation. It has been long known that antigen processing for MHC II-dependent presentation is mediated by endosomes/lysosomes (30), while MHC I-dependent antigen presentation is mediated by endoplasmic reticulum (ER). Autophagy also plays an important role in MHC II-dependent antigen presentation in B cells and dendritic cells, in which autophagosomes continuously merge with multi-vesicular compartments loaded with MHC II (Figure 1). Targeting of the influenza virus matrix protein-1 to autophagosomes by fusion of this protein with LC3, a marker of growing autophagosomes, strongly enhances the presentation of this protein to CD4^+^ T cells (31). Mice with conditional knockout (KO) of autophagosome-associated protein Atg5 in dendritic cells fail to mount sufficient CD4^+^ T cell priming after herpes simplex virus infection (32). Conditional KO mice lacking Atg5 in B cells have not been analyzed in this context, but would likely display a similar phenotype.

Protein citrullination is highly relevant to rheumatoid arthritis (RA), as RA patients display high levels of autoantibodies to citrullinated self-proteins (33). Citrullinated peptides, but not unmodified peptides, are presented to T cells by dendritic cells and macrophages through autophagy. In B cells, citrullinated peptides are also presented in a manner dependent on BCR engagement (e.g., triggered by anti-IgM) and autophagosome formation (which can be blocked by 3-methyladenine, an inhibitor of PI-3K and autophagy). Upon brief serum starvation, B lymphoma cells can also present citrullinated peptides through an Atg5- and autophagosome-dependent pathway (34).

### B Cell Activation and CSR/SHM

Intracellular membrane-associated proteins regulate activation and differentiation of B cells, in addition to their development and survival – pre-B cell development in the bone marrow and B-1 cell survival in the periphery are defective in mice with B cell conditional KO (*Cd19*-*cre*) of *Atg5* (35). Upon BCR engagement by antigen, the fast and extensive induction of autophagosomes, in which the antigen would be rapidly processed, can cause cell death (36). Autophagy-dependent cell death can be overcome by CD40 engagement for full B cell activation (36), suggesting a role of autophagy in controlling self-reactive B cell activation and autoimmunity.

In B cells activated by CD40 engagement or dual TLR/BCR engagement (37), Rab7 is upregulated, suggesting a role of Rab7 and Rab7^+^ late endosomes in peripheral antigen-dependent B cell differentiation. In conditional KO *Igh*^+^*^/C^*γ*^1-cre^Rab7^fl/fl^* mice, *Rab7* is ablated only in B cells undergoing *Igh^C^*γ*^1-cre^* Iγ1-Sγ1-Cγ1-*cre* transcription, as induced – like *Igh* germline Iγ1-Sγ1-Cγ1 transcription – by IL-4 in conjunction with CD40 or dual TLR/BCR engagement (38). These mice are normal in B and T cell development, but cannot mount T-independent or T-dependent class-switched IgG1 responses, while maintaining normal IgM levels. *Igh*^+^*^/C^*γ*^1-cre^Rab7^fl/fl^* B cells are normal in proliferation, survival, and plasma cell differentiation, as well as activation of the p38 kinase and ERK1/2 kinase pathways, but show defective CSR (38). This defect can be rescued by enforced expression of activation-induced cytidine deaminase (AID), which is essential for CSR and SHM. In addition, inhibition of Rab7 activity by a small molecule compound, CID 1067700 (39), reduces CSR and antibody responses in normal B cells/mice as well as autoantibody response and disease symptoms in lupus-prone MRL/*Fas^lpr/lpr^* mice (40). These findings, together with our demonstration that Rab7 mediates canonical NF-κB activation, as critical to AID induction, outline a novel role of Rab7 in signaling pathways that lead to AID expression and CSR, likely by promoting assembly of signaling complexes along mature endosomes. Such a role of Rab7 is consistent with the proximity of Rab7-containing mature late endosomes to the nucleus (Figure 1), as activated NF-κB would have a short path to reach genes, an advantage shared by ER membrane-mediated NF-κB activation, as occurring in BL41 B cells upon CD40 engagement and in Jurkat T cells upon TCR engagement (41). It would also be irrespective of the initial location of engaged immune receptors, e.g., on the plasma membrane (such as CD40, TLR1/2, and TLR4) or in endosomes (TLR7 and TLR9), and their trafficking pattern.

Lysosomes have been recently implicated to regulate signal transduction, serving as the “docking station” for mTOR in various cancer cells (42–44) and likely in B cells. Knock-in mice expressing a hypomorphic mTOR mutation and conditional KO mice with B cells mTOR deficiency are defective in germinal center formation and antibody responses, in concomitant with reduced AID expression and CSR (28). Pharmacological inhibition of mTOR kinase activity results in complicated phenotypes, likely due to non-redundant functions of two mTOR-containing complexes, mTORC1 and mTOC2 (28, 45).

### Plasma Cell Differentiation

The differentiation of activated B cells into antibody-secreting plasma cells is associated with changes in gene expression that lead to the loss of the B cell identity and the gain of protein secretion functions. Such changes are mediated by the master transcription factor Blimp-1 (B lymphocyte-induced maturation protein-1), as encoded by *Prdm1* (8). In plasma cells, ER membranes expand and enhance their capacity to fold nascent peptides, a process driven by X-box binding protein-1, to promote antibody secretion (46). Both differentiating plasmablasts (which still proliferate) and terminally differentiated long-lived plasma cells have high autophagic activities. In two independently generated mouse strains with conditional *Atg5* KO in B cells (both through *Cd19*-*cre*), antibody responses and generation of antigen-specific long-lived plasma cells are defective (47, 48), likely due to impairment in generation of plasma cells (47), plasma cell survival (48), or both – the ER-related secretion function of plasma cells, however, does not seem to be affected (48). In addition, autophagosomes have been suggested to serve as a platform in recruiting ERK and its activation (49), which plays an important role in Blimp-1 induction and plasma cell differentiation (50). Rab7 does not play a major role in the generation of plasma cells, but may help in maintaining plasma cell survival, perhaps also through NF-κB-dependent sustained expression of Blimp-1 (40). Thus, intracellular-associated proteins are important for plasma cell homeostasis and sustainable antibody production.

### Memory B Cell Differentiation

As recently shown, memory B cells specifically express high levels of autophagic activities and do so over time, i.e., low/no levels of autophagosome formation (LC3^+^ puncta) in naïve B cells, germinal center B cells, and newly formed memory B cells, but high levels in memory B cells isolated much later after the primary immunization (51). Atgs, such as *Ulk1*, *Atg14*, *Becn1* (encoding Beclin 1), *Atg5*, *Atg7*, and *LC3*, display similar expression patterns. Furthermore, Atg7 is dispensable for the initial generation of memory B cells, but critical for the long-term survival of memory B cells, likely by preventing apoptosis, as mice with B cell conditional KO of Atg7 (through *Cd19*-*cre*) can mount normal primary antibody responses, but much reduced secondary antibody responses and SHM upon re-challenging by antigen (51, 52). Finally, memory B cells express high levels of a fork-head family transcription factor FoxO3 and, to a lesser extent, FoxO1, consistent with the important role of these two factors in autophagy gene expression (53). How Atg7 and related intracellular membranes regulate apoptosis and perhaps other metabolic processes for the maintenance of memory B cells remains to be determined. Overall, intracellular membranes play an important role in peripheral antigen-dependent B cell differentiation. And, different membrane structures, likely through their associated proteins, specify different differentiation processes, e.g., Rab7 in CSR/SHM, Atg5 in plasma cell differentiation, and Atg7 in memory B cell differentiation (Figure 1).

## Regulation of B Cell Differentiation by Intracellular Membrane-Associated Proteins and Micrornas

### MicroRNAs Regulate Peripheral Antigen-Dependent B Cell Differentiation

MicroRNAs are endogenous small non-coding RNAs that regulate adaptive and innate immunity, including B cell differentiation (54, 55). They modulate gene expression through mainly post-transcriptional mechanisms, such as inhibiting translation of mRNAs and/or promoting their degradation after pairing with the 3′-untranslated region (3′-UTR) of the target mRNA (Box 1). A single microRNA can regulate multiple gene targets and, conversely, a single gene can be regulated by multiple microRNAs. Conditional KO of *Dicer*, which is essential for microRNA biogenesis, through *Cd19*-*cre* leads to impairment in the generation of follicular B cells with concomitant increase in marginal zone B cells (56). This, together with the higher Dicer expression in follicular B cells, supports the notion that selected microRNAs mediate this stage of B cell development. High titers of autoantibodies in female *Dicer* conditional KO mice suggest a role of microRNAs in preventing autoimmune diseases (56).

Box 1 **MicroRNAs biogenesis and function.**

MicroRNAs are a class of endogenous small non-coding RNAs, usually 19-23 nucleotide in length, that regulate gene expression through mainly post-transcriptional mechanisms. In microRNA biogenesis, a microRNA host gene is first transcribed by RNA polymerase II, giving rise to primary microRNA (pri-microRNA) transcripts. These are then processed by a complex composed of Drosha, an RNase III enzyme, and Pasha, a double-stranded RNA binding protein, to precursor microRNAs (pre-microRNAs), a stem-loop structure of 70-100 nucleotides. Pre-microRNAs are transported into the cytoplasm and cleaved by the Dicer RNase III. Resulting microRNA duplexes are incorporated into a RISC complex that also contains Ago. The “lead” functioning strand is guided to match the 3’-UTR of the mRNA target, while the “passenger” strand is degraded. A functional microRNA can pair perfectly or imperfectly with the microRNA recognition sites within mRNA 3’-UTR, leading to transcript degradation and translation inhibition, respectively.

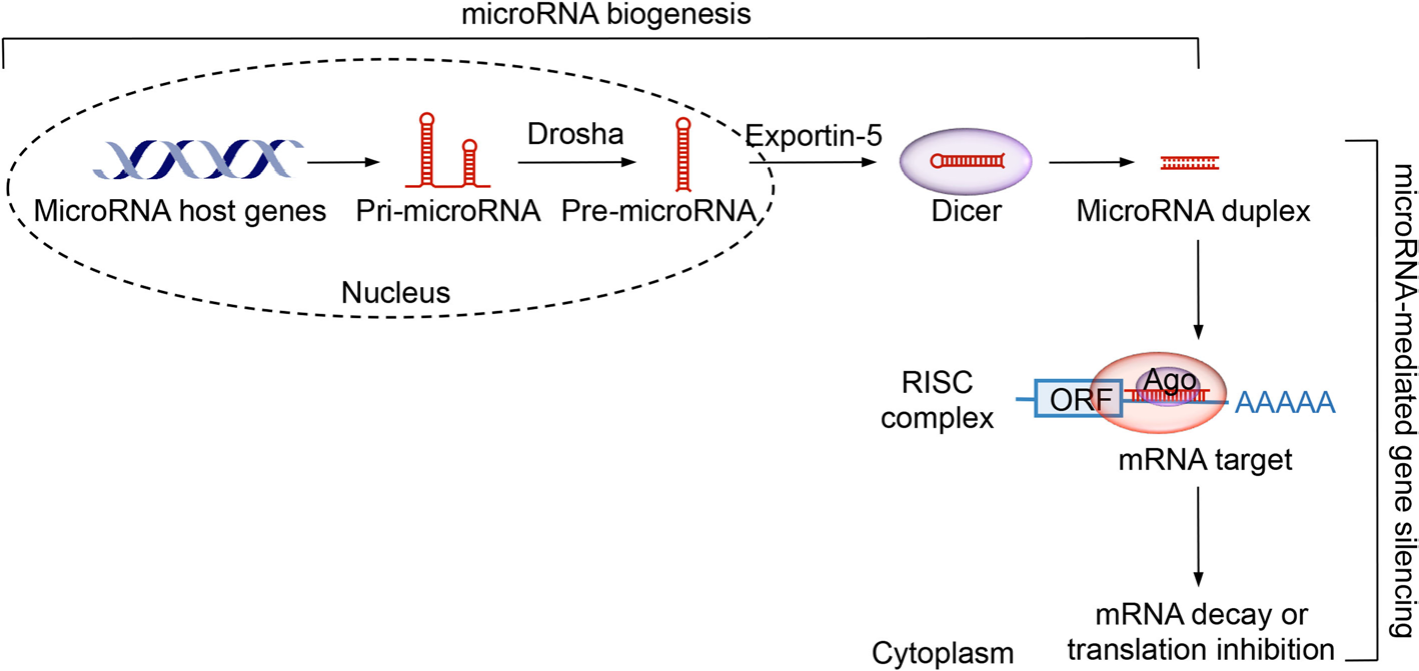

MicroRNAs regulate the germinal center reaction. As *Aicda* gene expression is restricted to B cells activated by T-independent or T-dependent stimuli, *Aicda*-*cre* mice have been widely used to generate activated B cell-specific KO of genes. Conditional KO of *Dicer* through *Aicda*-*cre* dampens germinal center formation, likely through elevated expression of Bim, a pro-apoptotic protein (57). *Dicer* KO B cells are also defective in proliferation and survival, leading to impairment in production of high-affinity class-switched antibodies and generation of plasma cells or memory B cells. The relevant microRNAs missing in these KO B cells would include miR-155, as this microRNA is highly expressed in germinal center B cells and miR-155 KO leads to defective germinal centers (58, 59).

MicroRNAs play vital roles in CSR and SHM. MiR-155 targets the *Aicda* 3′-UTR, as knock-in or transgenic mice with the miR-155-binding site mutated in *Aicda* 3′-UTR show higher level of AID expression with upregulation of CSR and SHM (60). MiR-181b can also target *Aicda* 3′-UTR to regulate AID expression and CSR. Accordingly, miR-181b expression is downregulated in activated B cells, thereby allowing accumulation of AID (61). MiR-210 has been shown to be upregulated in activated B cells and inhibit CSR, likely by downregulating CSR factors – miR-210-deficient mice produce high levels of autoantibodies (62). B cell differentiation into plasma cells is also regulated by microRNAs. Overexpression of miR-125b, which is expressed in centroblasts (activated germinal center B cells that are enlarged and proliferating), represses plasma cell differentiation and Ig secretion by targeting *Prdm1* (63). Finally, histone deacetylase inhibitors (HDI) upregulate miR-155, miR-181b, and miR-361 to silence *AICDA/Aicda*, as well as miR-23b, miR-30a, and miR-125b to silence *PRDM1/Prdm1* in human and mouse B cells (64), but not other CSR/SHM-related genes or microRNAs that are not known to regulate *AICDA/Aicda* or *PRDM1/Prdm1* (65). Importantly, HDI also abolish CSR/SHM and impair the class-switched high-affinity antibody responses in normal mice and pathogenic autoantibody response in lupus-prone mice (64).

### MicroRNAs Regulate Intracellular Membrane Functions

MicroRNAs regulate virtually all cellular pathways, including intracellular membrane functions, such as endocytosis (66), lysosome degradation (67), and autophagy (68). They would do so in cancer cells, stem cells, and B cells, which express relevant intracellular membrane structures. For instance, miR-509 can bind to the transcripts encoding Rab5C, one of the three Rab5 isoforms, thereby inhibiting Rab5C expression in pre-B acute lymphoblastic leukemia cells and resulting in a growth defect that can be rescued by Rab5C overexpression (69). Targeting of SUMF1, a cellular sulfatase activator, by miR-95 disrupts sulfatase activities, resulting in an accumulation of sulfated substrates in lysosomes that, in turn, impairs lysosome-mediated cargo degradation – miR-95-mediated lysosome dysfunction also results in defects in autophagy-mediated cargo degradation (67). Knocking down of miR-95 in cells isolated from patients with a severe lysosomal storage disorder called multiple sulfatase deficiency due to hypomorphic *SUMF1* mutations can increase SUMF1 protein levels, suggesting a potential therapeutic intervention for this disease (67). MiR-376b directly targets the 3′UTR of *ATG4C* and *BECN1* mRNA, thereby inhibiting Atg4C- and Beclin 1-dependent autophagy induced by starvation or rapamycin, and an antagomir of miR-376b increases Atg4C- and Beclin 1 expression and autophagy (70). Likewise, miR-20a and miR-106b suppresses ULK1 expression and autophagy, a process that can be de-repressed by nutrient (leucine) deprivation (71). Finally, systems biology approaches have led to the identification of several microRNAs (e.g., miR-130, miR-98, miR-124, miR-204, and miR-142) that can target lysosome-related proteins to modulate the autophagy–­lysosomal pathways (72).

### MicroRNAs Regulate Intracellular Membrane-Associated Proteins Involved in B Cell Differentiation

The miR-17–92 locus encodes a cluster of microRNAs (miR-17, miR-18a, miR-19a, miR-19b, miR-20a, and miR-92a) that play important roles in regulating immune functions (73). These microRNAs are essential for B cell development (74), but dispensable for mature B cell survival (75). Mature B cell-specific miR-17-92 KO mice are defective in producing class-switching IgG2c antibodies (75). Lymphocyte-specific overexpression of miR-17-92 increases proliferation and survival of B cells and germinal center B cell differentiation, eventually leading to lymphomagenesis (76). Among microRNAs in the miR-17-92 cluster, miR-17 targets transcripts encoding TBC1D2, which acts as a GTPase activating protein (GAP) of Rab7 to downregulates Rab7 activity (66) – TBC1D2 is also an effector of Rab7-GTP and would participate in the negative feedback-loop that controls Rab7 activities. MiR-17-dependent upregulation of Rab7 activities may explain, at least in part, the role of miR-17-92 in class-switched antibody responses. MiR-17 also targets Atg7 (77), suggesting a role of miR-17-92 in memory B cell differentiation, in addition to its inhibition of plasma cell homing to the bone marrow (75).

**Figure 2:**
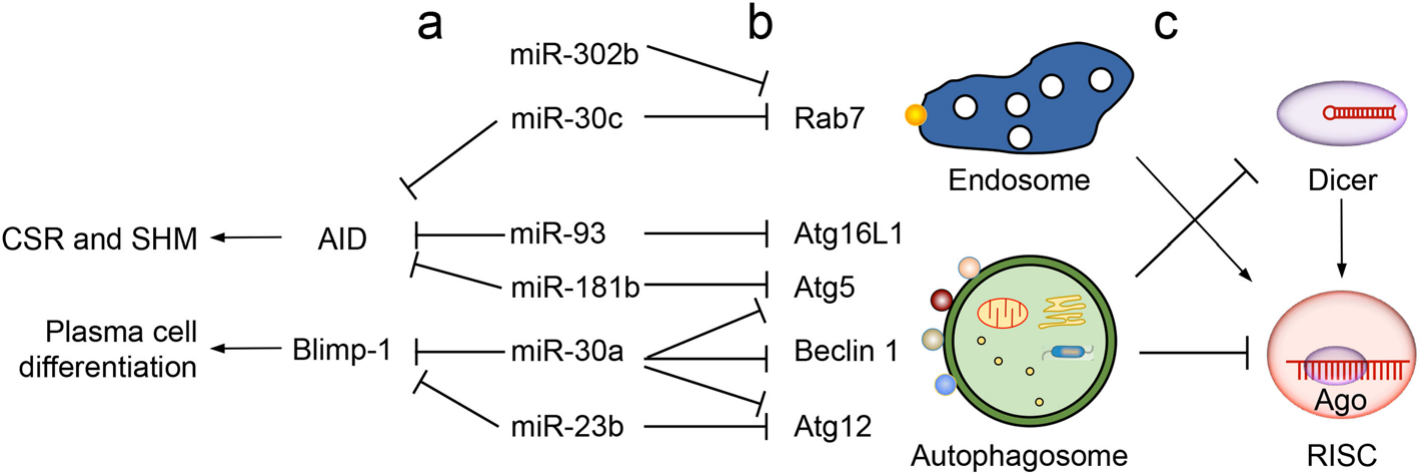
**Cross-regulation of intracellular membrane-associated proteins and microRNAs in B cell differentiation**. (a) Regulation of multiple intracellular membrane-associated proteins as well as AID (critical for CSR/SHM) and Blimp-1 (driving plasma cell differentiation) by a single microRNA. For example, miR-30a regulates Atg5, Beclin 1 and Atg12 in autophagy and Blimp-1, miR-30c regulates Rab7 (in the endosome pathway) and AID, as well, miR-93 and miR-181b regulate AID as well as Atg16L1 and Atg5, respectively. (b) Regulation of one intracellular membrane-associated protein by several microRNAs. For example, Rab7 is regulated by both miR-30c and miR-302b, and Atg12 is regulated by both miR-30a and miR-23b. (c) Regulation of microRNA activities by endosomes and autophagosomes. Endosomes provide a structure to support the assembly of RISC in microRNA-mediated gene silencing. Autophagosomes promote degradation of Dicer and Ago, thereby downregulating microRNA biogenesis and functions, respectively.

As we have recently reported (64), both miR-23b and miR-30a are upregulated by HDI in B cells to target the *Prdm1* 3′-UTR, thereby playing a role in mediating HDI repression of B cell differentiation into plasma cells (Figure 2). These microRNAs would also modulate plasma cell differentiation and/or functions by downregulating autophagic proteins, such as Atg5, Atg12, Beclin 1, and others factors (78–80) (Table 1). Another member of the miR-30 family, miR-30c, is predicted to target both Rab7 and AID, consistent with the notion that one microRNA can target multiple factors in the same pathway to maximize its influence on the outcome of that pathway (Figure 2, a). Other examples supporting this notion include miR-302b, which can target IRAK4 and likely Rab7, both of which are important for TLR-induced NF-κB activation (38, 81). In addition, miR-155 and miR-181b downregulate CSR by targeting AID. They may also inhibit plasma cell differentiation by virtue of their ability to target the 3′-UTR of transcripts encoding autophagic proteins, such as Atg5 and possibly Rheb and Rictor. MiR-93 targets the *AICDA* 3′-UTR and would regulate AID expression in activated B cells, as it does in the MCF7 breast cancer cell line (82), which, like several cancer cell lines, expresses elevated levels of AID, a potent DNA mutator and tumorigenesis factor (83). MiR-93 would also downregulate plasma cell differentiation by inhibiting autophagy and likely do so by targeting Atg16L1 (82), SQSTM1 (84), and possibly a battery of other autophagic proteins, such as ULK1, ATG14 and RB1CC1 (Table 1). Finally, consistent with the notion that a single factor can be regulated by multiple microRNAs, Rab7 would be regulated by both miR-30c and miR-302b (Figure 2, b), suggesting an (indirect) regulation of NF-κB induction and AID activation by these microRNAs.

**Table 1:** **MicroRNAs target intracellular membrane-associated proteins and factors critical for B cell differentiation and functions**.

MicroRNAs	Intracellular membrane-associated protein target	Specific factor target	Relevant B cell function	Reference
miR-17-92	TBC1D2, Atg7	Bim, Pten	B cell development, Germinal center reaction	(66, 73, 76, 84)
miR-23b	Atg12, Atg2B^a^, RAB11FIP2^a^	Blimp-1	Plasma cell differentiation	(64, 85)
miR-30^b^	Rab7^a^	AID^a^	CSR	(64, 79, 80)
	Atg5, Beclin 1, Atg12^a^	Blimp-1	Plasma cell differentiation	
miR-155	Rheb, Rictor	AID	CSR, SHM, Germinal center reaction	(60, 86, 87)
		SHIP-1		
miR-181b	Atg5	AID	CSR	(61, 88)
miR-93	Atg16L1, SQSTM1, Atg14^a^, RB1CC1^a^, ULK1^a^	AID	CSR	(82, 84, 89)
miR-302b	Rab7^a^, RABGAP1^a^, Rab9A^a^	IRAK4	NF-κB activation	(81)
miR-9	Atg14^a^, ULK2^a^	NF-κB	CSR	(90)
miR-10a	RB1CC1	BCL6	Germinal center reaction	(91, 92)
miR-146a	SQSTM1^a^	TRAF6	NF-κB activation	(93)

*^a^High confident targets predicted by miRDB (www.miRDB.org) (94)*.

*^b^Rab7 and AID are predicted to be targeted by miR-30c, with others being targets of miR-30a*.

As suggested by a recent study (95), intracellular membrane proteins would evade microRNA regulation by using mRNA transcripts with short 3′-UTRs for their production. Proteins can be encoded by mRNAs with alternative 3′-UTRs, the longer ones of which function as scaffold to interact with the RNA-binding proteins HuR and SET and facilitate the plasma membrane localization of membrane-associated proteins (95, 96) – HuR plays an important role in germinal center formation, CSR, and antibody responses, possibly though facilitating protein localization to the plasma membrane (97). When encoded by transcripts with shorter 3′-UTRs, which contain much fewer microRNA targeting sites and are less susceptible to microRNA-mediated silencing, these membrane-associated proteins would display a different subcellular localization, shifting from the plasma membrane to intracellular membrane structures.

Overall, the regulation of B cell differentiation and intracellular membrane-associated proteins by microRNAs would be deeply intertwined to achieve highly fine-tuned antibody responses.

## Conclusion and Perspectives

Intracellular membrane structures regulate, in addition to cellular homeostasis and basic metabolic processes, B cell differentiation in antibody responses and likely other cell type-specific functions. Different intracellular membranes are responsible primarily for distinct B cell differentiation processes, such as Rab7^+^ mature late endosomes in AID induction and CSR/SHM, Atg5^+^ autophagosomes in plasma cell differentiation and survival, and Atg7^+^ autophagosomes for memory B cell maintenance. Nevertheless, these lipid-containing micro-domains would also cross-talk, e.g., through their partially shared biogenesis pathways, thereby regulating multiple processes. Indeed, Rab7 would regulate plasma cell survival, in addition to its major role in CSR/SHM, and the collaboration of different membranes in regulating B cell differentiation is suggested by the role of ER in endosome biogenesis (98).

Specific functions of selected intracellular membrane-associated proteins would reflect their induction in differentiating B cells, as epitomized by the upregulation of Rab7 in activated B cells and elevated levels of Atg7 as memory B cells survive over the time. This notion emphasizes the importance of the profiling of expression of intracellular membrane-associated proteins and biogenesis of intracellular membrane structures in B cells toward our full understanding of B cell differentiation fate decision. As such, a comprehensive analysis of the role of intracellular membranes in signal transduction, which is ultimately responsible for gene regulation, is intriguing, as it would not only support the emerging paradigm that membrane organization specifies the signal output, but also reveal feedback-loop regulation mechanisms that contribute to the tight regulation of B cell differentiation.

As suggested by the critical dependence of endosomes on Rab5 expression levels (99), intracellular membranes are sensitive for their maintenance to the depletion of relevant factors, making microRNA, powerful regulators of intracellular membrane functions and, therefore, B cell differentiation. Interestingly, intracellular membranes would regulate B cell differentiation in part by modulating microRNA activities, as suggested by an important role of endosomes in promoting formation of an RNA-induced silencing complex (RISC) and downregulation of Dicer and Ago by autophagy (Figure 2, c). Ago2, a component of RISC, localizes to mature endosomes and RISC-bound mRNAs accumulate in GW bodies, which are discrete cytoplasmic foci associated with mature endosomes (100). Blocking endosomes maturation (by deletion of ESCRT complexes) results in loss of GW bodies and impairment in microRNA-mediated gene silencing, while accumulation of mature endosomes (through depletion of a tethering factor HPS4) enhances microRNA-mediated silencing (101, 102), all pointing to a positive role of endosomes in microRNA functions. By contrast, the autophagic pathway degrades Dicer and Ago that are not bound with any pre-microRNA and microRNA, respectively, in a manner dependent on the autophagy receptor NDP2, which interacts with LC3 (103). Dicer accumulates in cells deficient of the critical autophagy components Atg5, Atg6, or Atg7 (104), suggesting that B cell-specific conditional KO of *Atg5* and *Atg7* may display elevated levels of microRNAs that would, in turn, downregulate factors important for plasma cell differentiation (e.g., Blimp-1) and memory B cell maintenance.

Collectively, intracellular membrane structures and microRNAs would regulate important B cell processes. The reciprocal regulation of intracellular membranes and microRNAs would fine-tune at any time the gene expression necessary to achieve the specificity of B cell differentiation processes. Intracellular membranes can quickly regenerate upon replenishment of important relevant factors that were previously downregulated by microRNAs. This buffers the effect of a broad spectrum of aberrant molecular events, which frequently occur in highly proliferating B cells, and would play an important role in preventing disastrous overexpression of proto-oncogenes (such as Myc and BCL6) and the DNA mutator AID and, therefore, B cell lymphomagenesis (105–107).

## Conflict of Interest Statement

The authors declare that the research was conducted in the absence of any commercial or financial relationships that could be construed as a potential conflict of interest.

## Abbreviations

Ago, argonaute; AID, activation-induced cytidine deaminase; Atg, autophagy-related gene; BCR, B cell receptor; Blimp-1, B lymphocyte-induced maturation protein-1; CSR, class switch DNA recombination; ER, endoplasmic reticulum; GAP, GTPase activating protein; HDI, histone deacetylase inhibitors; Ig, immunoglobulin; KO, knockout; MHC II, major histocompatibility complex II; PI-3K, class III phosphatidylinositol 3-kinase; RA, rheumatoid arthritis; Rab, Ras-related in brain; RISC, RNA-induced silencing complex; SHM, somatic hypermutation; TCR, T cell receptor; TLR, toll-like receptor; VPS, vacuolar protein sorting.
